# Update on prostate cancer in black men within the UK

**DOI:** 10.3332/ecancer.2014.455

**Published:** 2014-08-28

**Authors:** Abeyna LC Jones, Frank Chinegwundoh

**Affiliations:** Department of Urology, Barts NHS Health Trust, The Royal London Hospital, Whitechapel Road, London E1 1BB, UK

**Keywords:** African Americans, African Caribbeans, Caucasian race, culture, ethnicity, literature review, risk, prostate, prostate-specific antigen, prostate cancer

## Abstract

There is a wealth of evidence which can be traced back to the African transatlantic slave trade indicating that black men have a higher risk of prostate cancer compared to other ethnic groups. Migration to Westernised countries may have had little effect on the incidence of prostate cancer in this ethnic group; however, current evidence indicates that there are several complex factors that may contribute to this risk.

Studies in the UK quote that black men are at 2–3 times the risk of prostate cancer in comparison to their Caucasian counterparts, with a 30% higher mortality rate. Caution should be taken prior to the interpretation of these results due to a paucity of research in this area, limited accurate ethnicity data, and lack of age-specific standardisation for comparison. Cultural attitudes towards prostate cancer and health care in general may have a significant impact on these figures, combined with other clinico-pathological associations.

This update summarises new contributory research on this subject, highlighting the need to increase awareness and understanding of prostate cancer amongst high-risk communities and to support further robust research in this area by nominating a lead in cancer and ethnicity studies within the National Health Service.

## Introduction

Prostate cancer (CaP) is a worldwide health burden with an estimated age-standardised incidence of 28 per 100,000 men [1]. Higher incidence rates are reported in nearly three quarters of developed countries, likely due to the use of widespread prostate-specific antigen (PSA) testing, routine histology from transurethral resections of the prostate and sophisticated diagnostic methods leading to the identification of early or latent cancers.

Incidence and mortality rates of CaP vary worldwide, partially due to disparities in the role of cancer registries and under-reporting, particularly in some developing countries. Despite this, there is a wealth of evidence demonstrating significant ethnic differences in mortality rates; the Caribbean population have the highest rates in the world (26.3 per 100,000) closely followed by sub-Saharan Africans (10 per 100,000), whereas Asians have the lowest (2.5 per 100,000) [1, 2]. CaP is estimated to be the leading cancer in incidence and mortality amongst African Caribbean (AC) men across the world and will continue to increase [2, 3], hence why this still remains an important health-care issue. 

CaP incidence and mortality variations between ethnic groups are likely due to genetic and environmental risk factors in combination with the local country health profile and robustness of health registries [4, 5]. The UK harbours a large and diverse migrant population including those originating from the Caribbean, Africa, and the Asian subcontinent. Each of these groups had a presence within the UK over several hundred years, later followed by a large influx of migrants in the post World War II era. There is evidence that black men and their descendants who live in developed countries like the UK have comparable incidences of CaP to those of their counterparts in their countries of origin [6–8].

Due to several reports of higher incidence rates of CaP in African Americans (AA) in comparison to Caucasians, a few similar studies have been published and reviewed in the UK illustrating a similar risk in British black men [3, 9–11]. A call for further research in this area was suggested, including further exploration of the comparison of rates between black men in developed countries and those living in their country of origin to determine whether migration plays an important role in the development of risk [9].

In this review, we summarise and discuss current evidence strengthening theories of a higher risk of CaP amongst the British black male community.

## Methods

This literature review was performed by conducting a systematic search of MEDLINE and PMC, including all articles up to November 2013. Keywords used for the search included ‘black men’, ‘prostate cancer’, ‘ethnicity’, ‘race’, ‘prostate cancer incidence’, and ‘prostate cancer risk’. All articles were reviewed and were included if they were relevant to the topic, published in English, and deemed to be of good quality. The references for each article were reviewed to identify further articles of relevance.

Institutions providing population-based data on prostate cancer statistics were reviewed. These included the National Cancer Intelligence Network, the National Cancer Institute, Cancer Research UK, and the Thames Cancer Registry. Previously unpublished data were retrieved from Public Health England.

## Origins of African Caribbeans in Western countries

The majority of Black men currently living in Western countries are descendants of those who originated from the transatlantic slave trade (TAST) between 1450 and 1900 where African slaves were exported mainly to the Americas, Europe, and the Caribbean [5]. Odedina *et al* comment that the burden of CaP amongst black men originating from West Africa clearly follows the path of the TAST [7].

ACs have settled in the UK for at least 300 years; however, the *Windrush generation* (referring to the first ship transporting a large number of Caribbean passengers to the UK in 1948) [12] is responsible for the mass introduction of immigrants from the British Empire who remain in the UK today. The AC population represents a diverse group of individuals who are mostly indigenous to Africa, yet also comprise Europeans and Asian-Indians, representing the heterogeneity of the gene pool due to generations of interbreeding.

Strong cultural habits and traditions remain in migrant communities, which has an unknown impact on health-seeking behaviours; therefore, caution should be used while interpreting data between ethnic groups, as possible reasons for disparities are likely multifactorial.

## Early studies identifying ethnic disparities in CaP

Some of the earlier studies comparing CaP incidence rates amongst ethnic groups reported the risk in AAs was at least twice that of their Caucasian counterparts [13, 14]. Conversely, sub-Saharan countries, including Nigeria, were labelled as a low-risk zone for CaP [14, 15]. Lower androgen levels and genetic predisposition were thought to be reasons for this low risk; however, subsequent studies challenged this notion reporting much higher incidence and mortality rates than previously thought [16, 17], also identifying that a high proportion of men with CaP in Nigeria had metastatic disease on presentation [18].

Glover *et al* presented similar findings in Jamaica, reporting an age-adjusted CaP incidence of 349 per 100,000 in Jamaican men in 1991 compared to an incidence of 249 per 100,000 in AAs in 1992 [19]. Of these patients, 42% presented with an abnormal digital rectal examination, and 16% presented with bone metastases. Subsequent increases in incidence from 1989 to 1994 were attributed to the increasing diagnostic use of PSA testing (7% to 48%). A later study using data from the Jamaica Cancer Registry in 1998 reported an age-specific incidence of 56.4 per 100,000 [20], initiating speculation that Glover *et al* ’s figures were actually much higher than expected.

The incidence of CaP amongst black men in developed countries is comparable to that of black men in developing countries [6–8], without taking into account the possible gross underestimation of incidence in the latter. Cancer registries are designed to systematically report relevant standardised data on a national level; however, not all countries, especially those in the developing world, maintain or possess a registry at similar standards.

Around the time sub-Saharan Africans were reported as having a low incidence of CaP, there was a lack of formal cancer registers, contributing towards under-reporting of the disease.

## CaP amongst black men in the UK

Despite a large AC contingent in the UK, there are few studies on the incidence and mortality of prostate cancer in this group in comparison with a high volume of studies originating in the US.

One of the first studies in the UK reporting ethnic variations in CaP was an audit of 359 newly diagnosed men (248 Europeans, 91 Afro-Caribbean, 20 South Asians) between 1999 and 2000 in North and East London [10]. The age-specific incidence rates for every age group were three times higher in ACs compared to Europeans. Similarly, there was a three times greater risk for men under the age of 70 in this group, as ACs would be diagnosed earlier (70.4 years) than Europeans (75.6 years) and South Asians (71.7 years). South Asians generally had the lowest risk, although notably this group was under-represented in this cohort. There was no significant difference in Gleason score, presenting PSA, and stage, although a modest increase was seen in the first two.

This study generated interest in this field and resulted in a collaboration known as the Prostate Cancer in Ethnic Subgroups (PROCESS) study group [9, 11, 21]. Between 1997 and 2001, the data were analysed from a larger retrospective cohort in London and Bristol, cities with a relatively high populous of black communities. There was an inclusion of 2140 incident cases with a demographic spread of 1315 (61.4%) white, 435 (20.0%) black Caribbean, 102 (4.8%) black African, 128 (6.0%) other ethnic groups, and 149 (7.0%) uncoded ethnicities.

Key findings included higher relative rates of prostate cancer for black men compared with white men, and this was even more marked for younger age groups. The age-standardised rates for white and black men were 56.4 and 166 per 100,000, respectively (*p* < 0.001 for all black men, *p* = 0.002 for black Caribbean men, *p* = 0.02 for black African men). Black men were around three times more likely to be diagnosed with prostate cancer than white men, and notably there was little difference between men of Caribbean or African origin.

These rates were compared with US age-specific rates of 283 per 100,000 for black men in 1999 from the USA’s Surveillance Epidemiology and End Results Programme (SEER), compared with 166 per 100,00 for UK black men (US white rate was 172 per 100,000). The higher incidence rates in the US are postulated to be due to a pronounced use of PSA testing leading to over diagnosis in those subjects [9].

Further publications by this group revealed no significant differences between ethnic groups and their access to health services, knowledge, delays to presentation or co-morbidities. PSA was, however, slightly higher amongst black men at diagnosis, they were more likely to be referred by a health-care professional, and lived in less affluent areas than their white counterparts [11].

The data derived from the Thames Cancer Registry reported 38,971 patients diagnosed with prostate cancer between 1998 and 2003 [22]. Black men had an age-adjusted incidence rate ratio of 2.5 compared to white, which remained to be even higher for men under the age of 60 (3.77). South Asians had an age-standardised incidence rate ratio of 0.69 compared to white men.

The National Cancer Intelligent Network (NCIN) released a report published in 2012 using broad age-standardised data from The Office of National Statistics between and 2006 and 2008. Black men had a 30% higher mortality rate compared to white men, with rates of 91.6 to 70.5 per 100,000, respectively. The mortality rate in those from the predominantly South-Asian subcontinent remained significantly lower than both caucasian and black men at 17.2 per 100,000; similar rates to that of their indigenous countries [23]. Unfortunately, due to the lack of sensitivity of broad age-standardised data, these figures could not be used for direct comparison to the US population.

Further data from the NCIN recorded an age-standardised mortality rate of 43 compared to 19 per 100,000 amongst black and white British men, respectively. Similar US data from SEER in 2008 report an age-standardised mortality rate of 49 compared to 21 per 100,000 for white and black US men, respectively, indicating that black men in both settings were just over double the risk of dying from prostate cancer. Notably, ethnicity was known for 99% of deaths recorded by the Office of National Statistics, nearly eliminating the statistical impact of ‘lost’ data for unidentified black individuals.

## CaP amongst black men outside the UK

A recent summary of available data from GLOBOCAN and SEER data in 2008, reported CaP as the most frequently reportable cancer diagnosed in black men in the US, Caribbean, and Sub-Saharan Africa (SSA). The incidence rate was highest amongst AAs (159.6 per 100,000), however the mortality rate was highest in the Caribbean (26.3 per 100,000 compared to 22.4 per 100,000 in the US) [24]. Comparatively, there was a low incidence of prostate cancer amongst SSAs (17.5 per 100,000), yet the mortality rate was five and four times that of AAs and Caribbeans, respectively. The significantly higher tumour stage, Gleason score, and presenting PSA in these SSA black men may account for these differences [24]. CaP continued to be the leading cancer for all these men for both incidence and mortality, excluding a higher mortality of lung cancer in AAs (49.1 compared to 22.4 per 100,000).

Chu *et al*’s data from the International Agency for Research on Cancer (IARC) and the National Cancer Institute SEER programme for 1973–2007 reported a disparity between incidence rates in East Africa (10–38 per 100,000) and West Africa (5–20 per 100,000); however, these remained lower than rates reported in AAs (80–195 per 100,000) [25].

Both papers acknowledge the limited data from African countries due to under-diagnosis and under-reporting, which may account for the comparatively low incidence rates in this region. The Africa Cancer Registry Network is a new initiative designed to address this issue, and help from future statistical studies on surveillance of all reportable cancers. In 2008, evidence from this registry supported CaP in Uganda as having the highest cumulative age-standardised incidence in 2008 of all male malignancies before Kaposi’s Sarcoma [26].

## Clinico-pathological associations with CaP in black men

Evidence relating to a postulated higher stage and PSA of prostate cancer at presentation remains inconclusive. A systematic review reports that black men present with CaP at an earlier age, with higher Gleason score, higher serum PSAs and a higher likelihood of locally advanced disease [27]. The UK PROCESS study reports similar findings with a raised presenting PSA in black men when age adjusted, however, Gleason scores and stages of localised disease remain comparable between black and white men. Black men also were more likely to undergo bone scans, MRI and CTs, possibly due to diagnosis at an earlier age [11].

Public Health England report stage data for 26% of known newly diagnosed cases of prostate cancer between 2008 and 2010, which illustrates comparably similar percentages of individuals in black and white ethnic groups at all three stages of presentation (see Table 1). Notably, there are a large number of individuals with unknown ethnicity, and a high proportion of unavailable stage data still remains, hence conclusions cannot be reliably made from these figures.

## The impact of behavioural and socio-economic factors within health-care interactions

Socio-economic status can be a significant issue relating to health-seeking behaviours. AAs are reported to be more likely to have a lower educational attainment, employment, and adequate health-care insurance, which may contribute to limited access to screening and health choices [27]. Black men within the UK were also more likely to be of a less affluent socioeconomic status [11], yet this had no effect on health access due to the widely accessible health-care structure of the NHS.

A well-designed meta-analysis of 48 studies concluded that health-care access, PSA screening, and comorbidities were not associated with a high risk of prostate cancer and mortality in black men [28]. AA men were offered fewer treatment options, or would opt for watchful waiting due to fear of side effects associated with curative treatment, resulting in a high likelihood of significant disease progression [27, 29–31]. Black men who opt for curative treatment were shown to be three times more likely to choose external beam radiotherapy over radical surgery [31].

Jack *et al* present findings from the UK Thames Cancer Registry reporting black and South Asian men were less likely to choose radical surgery or hormones compared to white men, however, the receipt of radiotherapy was similar amongst all ethnic groups [22].

In other ethnically diverse regions in the world, such as South Africa, black men were less likely to choose curative treatment, which were also attributed to, in part, by socio-economic reasons [32]. Reasons for these decisions are not always fully explored in the literature.

There is evidence to suggest that knowledge and attitudes towards prostate cancer may contribute towards presentation and treatment choices. A well-designed report by the BME Cancer Communities, summarises evidence illustrating a reduced awareness of cancer amongst black AC communities in the UK, delayed referrals, and poorer experience of the NHS cancer services [33]. Although a study has shown that GP referrals contribute towards 58% of the route to diagnosis for men with prostate cancer, there remains to be anecdotal evidence that GPs have refused PSA testing or neglected digital rectal examinations in this high-risk group [34]. These factors are possibly responsible for late presentation of prostate cancer in this ethnic group.

We can draw some ideas from a systematic literature review published in 2011 which compares and explores the knowledge and perceptions of prostate cancer amongst black men [35]. Themes include a perceived lack of doctor–patient communication, underestimation of risk of prostate cancer, higher level of fear, embarrassment, and loss of masculinity and prioritisation of work over health-care. Presentation with metastases, lower urinary tract symptoms, knowledge of CaP, and co-morbidities were comparable between black and white men in two UK papers [11, 22]. However, contrary to other reports, black men were more likely to be aware of their higher risk and more concerned about symptomatology. Despite this, they remained generally reluctant to see a doctor [11].

## The role of genetic and biological factors

The involvement of genetic and biological factors in the development of prostate cancer is extremely complex and a popular target for research.

The androgen receptor-signalling pathway is critical to the development of the normal prostate, benign hyperplasia, and prostate cancer. Androgen receptor expression is down-regulated in prostatic epithelial stromal tissue in black men [36] but has a high expression in malignant epithelium, which also correlates with a high risk of disease progression and aggressive CaP phenotype [37].

High testosterone levels in black men may play a factor in the pathogenesis of CaP [38]. A higher prevalence of polymorphism associated with the 5 alpha reductase type 2 gene and CYP3A4 protein, both of which regulate testosterone metabolism [39, 40] may result in a higher conversion rate of testosterone to DHT, conferring a higher risk of CaP, particularly of an aggressive subtype [41].

There is a wealth of evidence implicating 8q24 variant alleles in the development of CaP; some of which state that there is a higher frequency amongst those of African ancestry [42] and with earlier time to prostate cancer [43]. There are several other CaP susceptibility loci identified through genome-wide association studies, with inconsistencies between ethnic groups [44].

Obesity has demonstrated inconsistent links with CaP risk over the years. A recent systematic review of 23 prospective cohort studies concluded that obesity is a significant risk factor for CaP [45]. High central adiposity in comparison to global adiposity in AA men has been implicated in the development of CaP [46]. Other studies have suggested that diets high in animal fat and red meat cooked at high temperatures are associated with high advanced CaP risk in black men [47–49].

Dietary antioxidants including Vitamin E, selenium, Vitamin C, and beta-carotene have been studied for protective risks against prostate cancer, however, no convincing evidence for this has been reported [50, 51], and it is not clear whether there is any difference in intake of these substances between ethnic groups.

Notably, most of these studies have a relatively low AC contingent, hence their findings may not be generalisable.

## Discussion

There remains to be a deficiency of UK-based research on CaP risk amongst black men in comparison to the US. Until recently, PSA screening was widespread in the US, possibly contributing to apparently higher incidence rates amongst AAs in comparison to their British counterparts. Powell *et al* demonstrated the effect of targeted screening of CaP amongst AAs, revealing a high incidence of early, yet clinically significant prostate cancer in comparison to previous studies with Caucasian majority populations [52]. Future studies in the UK for high-risk individuals in ethnic minority groups may reveal similar findings if recruitment strategies are optimal.

AAs have been resident in the US for several generations, whereas most of the black British population are first- or second-generation migrants from countries in the Caribbean or Africa where CaP incidence is relatively high. Although the true effect of migration of risk remains unknown, we could postulate similar incidences of CaP with that of their country of origin, hence it remains possible that these individuals remain to be at a higher risk than other ethnic groups.

In comparison to the Kheirandish review published in 2011 [3], this update adds useful mortality data from the NCIN and Thames Cancer Registry, both of which are consistent with evidence from studies reporting a higher mortality rate amongst migrant black men and their descendants. Early data from Public Health England do not support theories that black men present with a higher stage of disease at presentation in comparison to their ethnic counterparts, yet it is likely that a high proportion of data for this ethnic group is lost in the ‘unknown’ ethnic category.

Data analysis for ethnicity is notoriously difficult. Crudely categorising ethnic groups can be misleading, especially with an increasingly culturally and ethnically diverse population. In the UK, prior to 2001, ethnicity coding was broad and inconsistent. From 2001, national standards according to the Department of Health mandated the use of 16 codes grouped under five headings, all of which are voluntary and self-assigned. This may assist in analysing data; however, those in ethnic minority groups may not want to declare an ethnicity, resulting in unaccounted individuals.

Studies and reports have also touched upon cultural factors and their relation to CaP risk in black men. There appear to be several more avenues of exploration to determine if or why black men are vulnerable to decision-making when it comes to presenting to health services, undergoing investigation, and treatment options. Further prospective cohort studies using common age-standardised statistical analyses and reporting of data are required, including detailed exploration of socio-cultural themes and attitudes related to CaP diagnosis and treatment amongst ethnic minority groups.

## Conclusion

CaP is no longer a taboo subject, however, further work is required to ensure that awareness in high-risk communities and amongst health-care professionals is addressed, fostering a relationship of understanding and trust. A suggestion of a lead in cancer and ethnicity within the NHS [33] is supported, with further consideration to extend this to the NCIN and National Institute for Cancer Health Research. Promoting self-declaration of ethnicity is essential for the interpretation of health data, which should be made publicly available and time relevant.

## Conflicts of interest

The author(s) declare that they have no conflict of interest.

## Figures and Tables

**Table 1.** CaP according to presenting stage data from Public Health England 2008–2010.

	Black	Asian	White	Other	Mixed	Not known
**Localised**	53%	47%	52%	40%	46%	61%
	(243)	(92)	(10,075)	(36)	(21)	(3,800)
**Locally advanced**	17%	24%	15%	20%	11%	17%
	(79)	(46)	(2,961)	(18)	(5)	(1,066)
**Advanced**	30%	29%	32%	39%	43%	22%
	(140)	(56)	(6,182)	(445)	(20)	(1,384)
**Total**	462	194	19,218	499	46	6,250
